# Distinct Clinicopathological Features and Prognosis of *Helicobacter pylori* Negative Gastric Cancer

**DOI:** 10.1371/journal.pone.0170942

**Published:** 2017-02-02

**Authors:** Kun-Feng Tsai, Jyh-Ming Liou, Mei-Jyh Chen, Chien-Chuan Chen, Sung-Hsin Kuo, I-Rue Lai, Kun-Huei Yeh, Ming-Tsan Lin, Hsiu-Po Wang, Ann-Lii Cheng, Jaw-Town Lin, Chia-Tung Shun, Ming-Shiang Wu, for the Taiwan Gastrointestinal Disease and Helicobacter Consortium

**Affiliations:** 1 Department of Internal Medicine, Gastroenterology and Hepatology Section, An Nan Hospital, China Medical University, Tainan, Taiwan; 2 Department of Internal Medicine, National Taiwan University College of Medicine and National Taiwan University Hospital, Taipei, Taiwan; 3 Department of Oncology, National Taiwan University College of Medicine and National Taiwan University Hospital, Taipei, Taiwan; 4 Department of Surgery, National Taiwan University College of Medicine and National Taiwan University Hospital, Taipei, Taiwan; 5 School of Medicine, Fu Jen Catholic University, New Taipei City, Taiwan; 6 Department of Pathology and Forensic Medicine, National Taiwan University Hospital, National Taiwan University College of Medicine, Taipei, Taiwan; Duke Cancer Institute, UNITED STATES

## Abstract

**Background:**

Whether the characteristics and prognosis of gastric cancer (GC) are different in patients with and without *Helicobacter pylori* (HP) remains controversial. The definitions of HP status in patients with atrophic gastritis but negative tests for HP are heterogeneous. We aimed to assess the impact of HP on the prognosis of GC using different definitions.

**Methods:**

From 1998 Nov to 2011 Jul, five hundred and sixty-seven consecutive patients with GC were included. HP status was determined by serology and histology. Patients with any positive test were defined as HP infection. Patients without HP infection whose serum pepsinogen (PG) I <70 ng/dl and PG I/II ratio < 3.0 were defined as atrophic gastritis and they were categorized into model 1: HP positive; model 2: HP negative; and model 3: exclusion of these patients.

**Results:**

We found four characteristics of HP negative GC in comparison to HP positive GC: (1) higher proportion of the proximal tumor location (24.0%, P = 0.004), (2) more diffuse histologic type (56.1%, p = 0.008), (3) younger disease onset (58.02 years, p = 0.008) and (4) more stage IV disease (40.6%, p = 0.03). Patients with negative HP had worse overall survival (24.0% vs. 35.8%, p = 0.035). In Cox regression models, the negative HP status is an independent poor prognostic factor (HR: 1.34, CI:1.04–1.71, p = 0.019) in model 1, especially in stage I, II and III patients (HR: 1.62; CI:1.05–2.51,p = 0.026).

**Conclusion:**

We found the distinct characteristics of HP negative GC. The prognosis of HP negative GC was poor.

## Introduction

Gastric cancer remains one of the leading causes of death worldwide[1]. Approximately70% of gastric cancer occurred in developing countries such as Eastern Asia[2]. *Helicobacter pylori (H*. *pylori)* is an important causal factor of non-cardiac gastric cancer. The attributable fraction of *H*. *pylori* for gastric cancer has been estimated to be about 70%[3], which indicates that about 70% of gastric cancer could be prevented through eradication of *H. pylori[4–6]*. Also, the association of H. pylori eradication with a reduced incidence of gastric cancer was demonstrated in a meta-analysis study[7]. Nevertheless, gastric adenocarcinoma is a heterogeneous disease. About 30% of gastric cancers are not related to *H*. *pylori* infection[3]. Demographic feature, life style, high salt with nitrate intake, race and genetic variables contribute to the heterogeneity[8–12]. Epstein–Barr virus infection associated lymphoepithelioma-like carcinoma is another entity which causes about 5% of gastric cancer[13].

The proportion of HPNGC among gastric cancer patients varied from 0.66% to 24.6% in previous reports[14–16]. Whether the clinicopathological features and prognosis of *Helicobacter pylori* negative gastric cancer (HPNGC) are distinct to that of *Helicobacter pylori* positive gastric cancer (HPPGC) also remains controversial. Whereas some studies showed that patients with HPNGC had higher proportion of the proximal tumor location, more diffuse histologic type and younger age of disease onset as compared to those of HPPGC[14–17], other studies failed to show the associations. The contradictory results might be attributed to the differences in the prevalence of *H*. *pylori* infection in the countries where these studies were conducted[15,18,19]. Another explanation might be the different definitions of *H*. *pylori* negative status in patients with gastric cancer, especially for those with coexisting atrophic gastritis. *H*. *pylori* might not be detected using serology, histology, urea breath test or culture in patients with *H*. *pylori* associated atrophic gastritis[20–21]. Some of the previous studies categorized these patients as HPNGC, whereas others categorized them as HPPGC[16]. Some studies excluded patients with co-existing atrophic gastritis[15].

Therefore, we aimed to assess whether the clinicopathological features and prognosis of HPNGC are distinct to HPPGC using different definitions of *H*. *pylori* negative status. In the present study, the serum pepsinogen method was used to identify the co-existing atrophic gastritis[22–24].

In Eastern countries, the atrophic gastritis which was caused by H. pylori infection would drive H. pylori out of the gastric mucosa while the atrophy progressed[22]. Therefore, the patients with serological atrophic gastric phenotype in whom all the tests for *H*. *pylori* were negative might be classified into either positive H. pylori status or negative H. pylori status. For these patients, we performed three different models in the statistical analyses to find the influence of the misclassification. We categorized them into model 1: HPPGC; model 2: HPNGC; and model 3: exclusion of these patients. We analyzed the impact of *H*. *pylori* status on the clinicopathological features and outcomes of gastric cancer using the above definitions in the statistic models. We expected that the worst scenario in model 2 which might have most misclassified cases showed the poor prognostic effect of the negative H. pylori status.

## Material and Method

### Patients

Patients with histological proven gastric adenocarcinoma who were aged 20 years and older were eligible for inclusion. Patients with (1) histological proven lymphoepithelioma-like carcinoma; (2) remnant stomach gastric adenocarcinoma; and (3) history of *H*. *pylori* eradication prior to the diagnosis of gastric cancer were excluded from this study. From 1998 Nov to 2011 Jul, five hundred and sixty-seven consecutive patients who received standard treatment or best supportive care in National Taiwan University Hospital were enrolled in this hospital-based cohort study. The age range of patients were 22–92 years old. The written informed consents for all the participations in the study were obtained. The sera were prospectively collected at the time of diagnosis prior to endoscopic examination. Demographic data, including age, gender, comorbid illness, and life style factors were also recorded.

### Treatment and follow-up

All the patients received standard treatment according to the gastric cancer stage in National Taiwan University Hospital. Surgery consisted of subtotal gastrectomy, total gastrectomy with extended lymph-node dissection. The histological types, stage, and types of treatment (surgery, chemotherapy, or others) in patients with gastric cancer were recorded. The histology subtypes were classified according to Lauren’s classification (diffuse type, intestinal type, and mixed type). We categorized the location of gastric cancer into (1) cardia; (2) antrum and corpus; and (3) cardia and body. The stage of gastric cancer was classified according to the 7^th^ edition of American Joint Committee on Cancer (7^th^ AJCC) staging. The patients were followed up every three to six months by medical oncologists or surgical oncologists. Follow-up studies included blood cell count and biochemistry, chest X-ray, computed tomography, upper gastrointestinal series and esophagogastroduodenoscopy. Our study was approval by the ethics committee of National Taiwan University Hospital (201402057RINA).

### *H*. *pylori* status determination

*H*. *pylori* status was determined by serology (ELISA based assay, R-Biopharm AG, Germany) and histology in all eligible cases. Any positive of the two tests was defined as positive *H*. *pylori* status. The serum pepsinogen (PG) I and II levels were determined using commercially available kits (Eiken. Chemical Co., Ltd., Tokyo, Japan). Patients with a pepsinogen I level ≤ 70 ng/mL and a PGI/II ratio ≤3 were defined as atrophic gastritis[15]. For patients with serological atrophic gastric phenotype in whom all the tests for *H*. *pylori* were negative, we categorized them into model 1: *H*. *pylori* positive; model 2: *H*. *pylori* negative; and model 3: exclusion of these patients in the Cox proportional-hazards regression analysis.

### Statistical analyses

Pearson Chi-squared test was used to investigate the associations between *H*. *pylori* status and categorical variables. Two tailed Student’s t test was used to compare the continuous variables. The date that the gastric cancer was diagnosed pathologically were recorded. We assessed overall survival (OS) in all eligible cases that were alive, regardless of relapse status. The event in the overall survival analysis was defined as all-causes mortality. Relapse-free survival in all cases that were alive without local or distant recurrence was analyzed. The log-rank test, Kaplan-Meier survival analyses, and Cox proportional-hazards regression models were used to assess the impact of the variables on survival. The Stata 12 (64-bit) software was used for statistical analyses. A p-value of less than 5% was considered as statistically significant.

## Result

### Clinicopathological features of gastric patients with and without *H*. *pylori* infection

Of the five hundred and sixty-seven patients with gastric cancer, four hundred and thirty-five patients (76.7%) were positive for *H*. *pylori* infection. One hundred and thirty-two patients (23.3%) with gastric cancer were negative for *H*. *pylori* infection. Of these patients with negative *H*. *pylori* infection, fifty-seven patients had gastric atrophy which was determined by serum pepsinogen method. Table 1 showed the demographic characteristics of the three categories. The proportion of gender showed no statistical difference. The mean age of disease onset of negative *H*. *pylori infection* patients without gastric atrophy and positive *H*. *pylori* infection patients were 58.02 and 62.18 years, respectively (p = 0.008). The proportion of the proximal tumor location was higher in negative *H*. *pylori* infection patients without gastric atrophy than positive *H*. *pylori* infection patients (24.0% versus 11.2%, P = 0.004). There was more diffuse histologic type in negative *H*. *pylori* infection patients without gastric atrophy than positive *H*. *pylori* infection patients (56.1% versus 36.0%, p = 0.008). Our result showed more A blood group in positive *H*. *pylori* infection patients than negative *H*. *pylori* infection patients without gastric atrophy (38.4% versus 24.0%, p = 0.05). We also found that there were more patients with stage IV disease in negative *H*. *pylori* infection patients without gastric atrophy compared to positive *H*. *pylori* infection patients (40.6% versus 26.5%, p = 0.03). The pepsinogen I level and the pepsinogen I/II ratio were 83.1 +/- 19.17 ng/dl and 5.89 +/- 1.35 in *H*. *pylori-negative* patients who had no gastric atrophy. The pepsinogen I level and the pepsinogen I/II ratio were 69.1 +/- 6.32 ng/dl and 4.09+/- 0.27 in *H*. *pylori-positive* patients. The pepsinogen I level and the pepsinogen I/II ratio were 46.2+/- 8.48 ng/dl and 3.22+/- 0.64 in *H*. *pylori-negative* patients who had gastric atrophy. The mean age of disease onset of *H*. *pylori-negative* patients with gastric atrophy and *H*. *pylori-positive* patients were 69.16 and 62.18 years, respectively (p = 0.0003). There was more intestinal histologic type in *H*. *pylori-negative* patients with gastric atrophy than *H*. *pylori-positive* patients (56.1% versus 39.3%, p = 0.006). There were more patients with stage IV disease in *H*. *pylori-negative* patients and gastric atrophy compared to *H*. *pylori-positive* patients (42.1% versus 26.5%, p = 0.03).

**Table 1:** Characteristics of patients with gastric cancer assessed by *H*. *pylori* status.

Characteristics	*HP positive(A)*	*HP negative(B)*	*HP negative(C)*	P value	
	(N = 435)	Without atrophy (N = 75)	With atrophy (N = 57)	A vs. B	A vs. C
Age at diagnosis	62.18 +/- 13.63	58.02 +/- 15.70	69.16 +/- 14.36	0.008	0.0003
Gender				0.75	0.60
Men	265 (60.9%)	47 (62.6%)	36 (63.1%)		
Women	170 (39.1%)	28 (37.4%)	21 (36.9%)		
Blood type				0.05	0.35
A type	167 (38.4%)	18 (24.0%)	19 (33.3%)		
Non A type	268 (61.6%)	57 (76.0%)	38 (66.7%)		
Tumor location				0.004	0.04
Proximal location	49 (11.2%)	18 (24.0%)	12 (21.0%)		
Distal location	386 (88.8%)	57 (76.0%)	45 (79.0%)		
Resection modality				0.45	0.10
ESD	6 (1.8%)	0 (0%)	1 (2.8%)		
Subtotal gastrectomy	251 (78.9%)	33 (75%)	12 (34.2%)		
Total gastrectomy	61 (19.3%)	11 (25%)	22 (63.0%)		
Tumor resection				0.85	0.59
R0	297 (93.3%)	40 (90.9%)	32 (91.4%)		
R1	20 (6.4%)	3 (6.8%)	3 (8.6%)		
R2	1 (0.3%)	1 (2.2%)	0 (0%)		
Histological analysis					
Lauren classification				0.008	0.006
Diffuse type	157 (36.0%)	42 (56.1%)	16 (28.0%)		
Intestinal type	171 (39.3%)	22 (29.3%)	32 (56.1%)		
Mixed type	107 (24.7%)	11 (14.6%)	9 (15.9%)		
Invasive depth				0.62	0.10
T1	93 (22.2%)	14 (20.0%)	6 (11.5%)		
T2	34 (8.1%)	6 (8.5%)	4 (7.8%)		
T3	87 (20.6%)	18 (25.7%)	15 (28.8%)		
T4	207 (49.1%)	32 (45.7%)	27 (51.9%)		
Nodal metastasis				0.90	0.38
N0	138 (32.7%)	20 (29.1%)	12 (23.1%)		
N1	55 (13.0%)	11 (15.9%)	8 (15.3%)		
N2	104 (24.7%)	15 (21.7%)	12 (23.1%)		
N3	124 (29.4%)	23 (33.3%)	20 (38.5%)		
Stage (AJCC 7^th^)					
I	104 (24.6%)	14 (21.8%)	7 (12.2%)	0.38	0.06
II	61 (14.5%)	7 (10.9%)	4 (7.2%)	0.66	0.15
III	145 (34.4%)	17 (26.7%)	22 (38.5%)	0.18	0.26
IV	112 (26.5%)	26 (40.6%)	24 (42.1%)	0.03	0.03

N: number; ESD: endoscopic submucosal dissection; AJCC 7^th^: 7^th^ edition of staging system of the American Joint Committee on Cancer.

### Survival in gastric patients with and without *H*. *pylori* infection- Kaplan Meier curve

**Fig 1:**
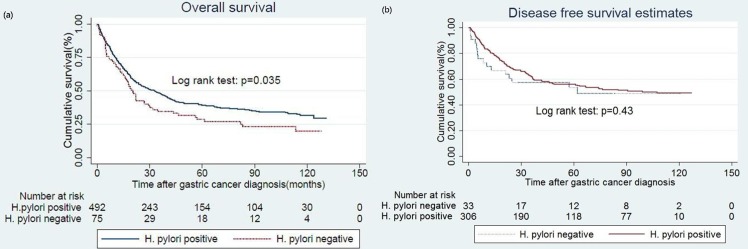
Influence of H. pylori status on overall survival and relapse free survival. Overall survival (a), relapse free survival (b).

**Fig 2:**
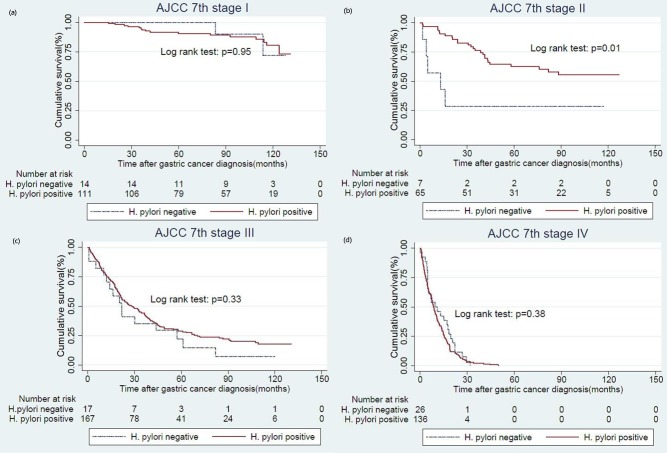
Influence of H. pylori status on overall survival according to AJCC 7th stages. stage I (a), stage II (b), stage III (c), stage IV (d).

**Fig 3:**
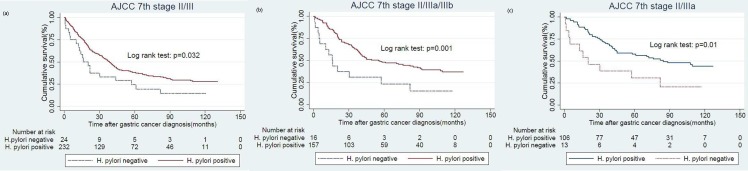
Influence of H. pylori status on overall survival according to AJCC 7th stages. stage II/III (a), stage II/IIIa/IIIb (b), stage II/IIIa(c).

In high *H*. *pylori* prevalence area, atrophic gastritis was highly associated with past *H*. *pylori* infection[15]. Therefore, we classified patients with negative *H*. *pylori* infection and atrophic gastritis as past *H*. *pylori* infection in model 1. At the time of analysis (Jan 1, 2015), the cancer related death happened in three hundred and sixteen(64.2%) of the HPPGC patients and in fifty seven(76.0%) of the HPNGC patients. In Kaplan Meier survival analysis, the long term OS rate was 35.8% in HPPGC patients and 24.0% in HPNGC patients (log-rank test, p = 0.035; Fig 1A). In Kaplan Meier survival analysis, the long term relapse free survival rate was 52.6% in HPPGC patients and 48.5% in HPNGC patients (log-rank test, p = 0.43; Fig 1B). For patients with stage I gastric cancer, there was no significant difference in long term OS between HPPGC and HPNGC (log-rank test, p = 0.95; Fig 2A). For patients with stage II gastric cancer, the long term OS rate was 28.5% in HPNGC and was 60.0% in HPPGC (log-rank test, p = 0.01; Fig 2B). For patients with stage III gastric cancer, the long term OS rate was 11.8% in HPNGC and was 22.7% in HPPGC (log-rank test, p = 0.33; Fig 2C). For patients with stage IV gastric cancer, there was no significant difference in long term OS (log-rank test, p = 0.38; Fig 2D). In the subgroup analysis, there was detectable statistical significant in patients with stage II or III gastric cancer (log-rank test, p = 0.032; Fig 3A). For patients with stage II, IIIa or IIIb gastric cancer, the long term OS showed statistical significance (log-rank test, p = 0.001; Fig 3B). For patients with stage II or IIIa gastric cancer, the long term overall survival was also statistically significant (log-rank test, p = 0.01; Fig 3C).

### Analysis of prognostic factors of gastric cancer

In model 1, we classified those with negative *H*. *pylori* tests who had gastric atrophy as HPPGC. Cox proportional-hazards regression model showed that negative *H*. *pylori* status was an independent prognostic factor for poor OS (hazard ratio, 1.34; 95% confidence interval, 1.04–1.71; p = 0.019). Age, invasive depth, nodal metastasis, non-curative resection, proximal tumor location and diffuse histologic type were all independent prognostic factors for poor prognosis (Table 2). In model 2, we classified these patients as HPNGC (Table 2). In model 3, we excluded these patients from our analysis (Table 2). In model 2, negative H. pylori status presented no significance of the poor prognostic effect. In analysis of patients with stage I,II,III using the model 1, we found that negative H. pylori status was the independent prognostic factors for poor prognosis (hazard ratio 1.62; 95% confidence interval, 1.05–2.51; p = 0.026) (Table 3).

**Table 2:** Predictive factors for overall survival in multivariate Cox proportional-hazard analysis in patients with gastric cancer.

	Model 1 (N = 567)		Model 2 (N = 567)		Model 3 (N = 510)	
Prognostic factors	HR (95% C.I)	P value	HR (95% C.I)	P value	HR (95% C.I)	P value
**Age**	**1.01(1.00–1.02)**	**<0.001**	**1.01(1.00–1.02)**	**<0.001**	**1.01(1.00–1.02)**	**<0.001**
**Negative *H*. *pylori* status**	**1.34 (1.04–1.71)**	**0.019**	**1.19 (0.95–1.50)**	**0.12**	**1.28(0.98–1.67)**	**0.06**
**Diffuse histologic type**	**1.19 (1.03–1.36)**	**0.013**	**1.19(1.04–1.37)**	**0.011**	**1.15(0.99–1.33)**	**0.06**
**Proximal tumor location**	**1.32 (1.00–1.73)**	**0.044**	**1.34(1.03–1.76)**	**0.033**	**1.28(0.95–1.72)**	**0.09**
**Invasive depth (T3/4 vs. T1/2)**	**4.02 (2.39–6.76)**	**<0.001**	**4.05(2.41–6.82)**	**<0.001**	**4.19(2.42–7.24)**	**<0.001**
**Nodal metastasis (N1/2/3 vs. N0)**	**2.98 (2.08–4.25)**	**<0.001**	**2.92(2.05–4.16)**	**<0.001**	**3.10(2.13–4.51)**	**<0.001**
**Non-curative resection**	**2.59 (2.05–3.27)**	**<0.001**	**2.54(2.01–3.21)**	**<0.001**	**2.61(2.03–3.35)**	**<0.001**

Patients with atrophic gastritis and negative *H*. *pylori* serology and histology (group C in Table 1) were classified into Model 1: *H pylori* positive GC; Model 2: *H*. *pylori* negative GC; and Model 3: exclusion from analysis.

All the analyzed variables including age, negative *H*. *pylori* status, invasive depth, nodal metastasis, Non-curative resection, diffuse histologic type, proximal tumor location were listed in the Table 2.

**Table 3:** Predictive factors for overall survival in multivariate Cox proportional-hazard analysis of patients with stage I, II or III gastric cancer.

Prognostic factor	HR (95% C.I)	P value
**Age**	**1.01 (1.01–1.03)**	**<0.001**
**Negative H. pylori status**	**1.62 (1.05–2.51)**	**0.026**
**Invasive depth (T3/4 vs. T1/2)**	**4.29 (2.46–7.46)**	**<0.001**
**Nodal metastasis (N1/2/3 vs. N0)**	**3.03 (2.05–4.46)**	**<0.001**
**Diffuse histologic type**	**1.12 (0.85–1.49)**	**0.007**
**Proximal tumor location**	**1.76 (1.16–2.67)**	**0.002**
**Non curative resection**	**2.14 (1.32–3.45)**	**<0.001**

All the analyzed variables including age, negative H. pylori status, invasive depth, nodal metastasis, Non-curative resection, diffuse histologic type, proximal tumor location were listed in the Table 3.

## Discussion

In our study, we found four characteristics of HPNGC in comparison to HPPGC, including (1) higher proportion of the proximal tumor location (24.0%, p = 0.004); (2) more diffuse histologic type (56.1%, p = 0.008); (3) younger disease onset (58.02 years, p = 0.008) and (4) more stage IV cancer (40.6%, p = 0.03). The novelty and strength of this study was that we analyzed the impact of *H*. *pylori* status on survival using three different definitions of *H*. *pylori* infection in patients with atrophic gastritis and negative serology test and histology for *H*. *pylori*. We found that negative *H*. *pylori* infection was an independent worse prognostic factor of gastric cancer by using the pepsinogen method. The poor prognostic effect of negative *H*. *pylori* status was particularly significant in patients with stage I, II and III disease.

The proportion of HPNGC varied from 10% to 40% among GC in the literature[15–17]. The prevalence and the differences in the definition of *H*. *pylori* status might contribute to the heterogeneous results. In western countries where the average prevalence of *H*. *pylori* was low, the proportion of HPNGC was higher and ranged from 13.8% to 24.6%[14,16]. In studies that classified patients with atrophic gastritis and negative tests for *H*. *pylori* as past *H*. *pylori* infection, the proportion of HPNGC tended to be lower. These studies also tended to report higher proportion of the diffuse type of gastric cancer in HPNGC patients[14]. Meanwhile, one previous study showed that the atrophic gastritis was caused by both the aging process and *H*. *pylori* infection[25]. It partially explained that the HPNGC patients were younger than HPPGC ones in Eastern Asia such as Japan[15]. In our study, we found that the proportion of HPNGC would be 13.2%, 23.2%, and 14.7% when patients with atrophic gastritis and negative *H*. *pylori* test were classified as HPPGC, HPNGC, and exclusion from analysis, respectively. The demographic result of H. pylori negative with atrophy patients showed elder disease onset (69.16 versus 62.18, p = 0.0003), more intestinal histologic type (56.1% versus 39.3%, p = 0.006), and more stage IV cancer (42.1% versus 26.5%, p = 0.03). Previous study have shown that the progressive atrophic gastritis which was caused by both the aging process and H. pylori infection could drive H. pylori out of the gastric mucosa[25]. Therefore, we assumed that negative H. pylori gastric cancer patients with atrophy should belong to HPPGC. The intestinal-type GC development is through an adenoma carcinoma sequence in the elder patients[26]. This might explain that the gastric cancer patients with negative H. pylori and mucosal atrophy in our study showed elder disease onset age and more intestinal histologic type. Previous study showed that gastric cancer in the elderly might contain more stage IV cancer[27]. The elder disease onset might be the reason that the patients with negative H. pylori infection and atrophy had more stage IV cancer. We also confirmed a higher proportion of diffuse type GC in patients with HPNGC by using the pepsinogen method.

Our result (24.0% vs. 11.2%, p = 0.004) was in agreement with prior studies in Italy (27% vs. 13%, p = 0.034) and Korea (25.6% vs. 12.8%, p = 0.014) that the proportion of proximal tumor location was higher in HPNGC[14,28]. One of the distinct pathogenesis of gastric cardia cancer is similar to that of esophageal adenocarcinoma which might arise from reflux-induced intestinal metaplasia change[29]. This carcinogenesis might be attributed to the high salt diet, metabolic effect or smoking and is independent of *H*. *pylori* infection[30–31]. Kwak HW et al. reported that patients with HPNGC had a higher frequency of distant metastasis (9.3% vs. 1.2%, p = 0.003)[32] and previous studies demonstrated that patients with HPNGC had a trend of the higher frequency of distant metastasis[14,28]. In our study, there were more stage IV cases in patients with HPNGC which indicated that HPNGC might have a more aggressive behavior. Our finding also showed that non-A blood type was dominant in investigating HPNGC patients (76.0%, p = 0.05). Previous studies supported that patients with blood group A were more susceptible to atrophic gastritis and in turn the higher risk of subsequent development of HPPGC[33–34].

Whether the *H*. *pylori* status is an independent poor prognostic factor of GC remains conflicting in the published literature[14,16,28]. The conflicting results might be attributed to the differences in the definition of *H*. *pylori* status and relatively small sample size in some of the previous studies[35–37]. In the present cohort study, we included larger sample size with longer follow-up period. More importantly, we used the pepsinogen method in our model and confirmed that the negative *H*. *pylori* status is an independent factor for poor prognosis. The definition of *H*. *pylori* status in some Westerns studies was similar to our model 2. However, their studies revealed negative *H*. *pylori* status was a poor prognostic factor[14,16]. In Taiwan, *H*. *pylori* prevalence was high and gastric mucosal atrophy is prevalent, whereas in the Western country *H*. *pylori* prevalence was average. Without the pepsinogen method, we cannot find the significance of poor prognostic effect in the negative *H*. *pylori* status. Whether the impact of *H*. *pylori* status on survival varied according to stage of GC was also conflicting in the literature. Some studies reported that the negative *H*. *pylori* status was a poor prognostic factor in early stages as well as advanced stages[14,28,38]. However, Georgios et al.[16] reported that the effect of negativity *H*. *pylori* status was more pronounced in patients with early stage cancer (HR 2.00 CI1.22–3.27, p = 0.0057) but not in patients with stage III or IV disease. Our scenario was to find certain cancer stage that had the worst prognostic effect. In our study, we further analyzed the prognostic effect in stage I, II and III cancers which showed worse hazard ratio 1.62 (95% confidence interval, 1.05–2.51; p = 0.026) in the negative H. pylori status than the HR 1.34 (95% confidence interval, 1.04–1.71, p = 0,019) in all stage cancers. The reason might be that the overall survival showed no difference between HPPGC and HPNGC in stage IV cancers (Fig 2). The clinical implication of this finding is that more aggressive treatment and surveillance strategies might be necessary in patients with HPNGC compared to those with HPPGC especially in stage I, II and III cancer.

Patients with HPNGC contained unfavorable prognostic factors including diffuse histologic type, proximal tumor location and stage IV cancer. However, the prognostic effect of HPNGC remained worse than that of HPPGC after adjustment for these variables, which indicated more aggressive biologic behavior of HPNGC compared to HPPGC. Diffuse-type GC develop through the de novo pathway in the younger patients. On the contrary, the intestinal-type GC developments are through a multistep process in the elder patients[26]. We postulated that the leading genetic or epigenetic alteration in HPNGC such as E-Cadherin (E-CDH) and genetic microsatellite instability (MSI) might be associated with the characteristics of HPNGC which had the manifestation of younger disease onset and diffuse histologic type. In our cohort, HPNGC had more stage IV stage which implied that HPNGC might have higher metastatic potency. The genetic or epigenetic alteration of E-Cadherin (E-CDH) and genetic microsatellite instability (MSI) might also cause regional or distal metastasis[39–41]. Furthermore, the matrix metalloproteinases (MMPs) that change the structure of vascular basement membrane promote cancer cell invasion and metastasis[42]. Angiogenic factors such as vascular endothelial growth factor (VEGF) that produced by tumor cell might induce neovascularization which are highly associated with metastasis[43]. We hypothesized that these metastatic related genes might be associated with HPNGC development. However, more studies are warranted to delineate the molecular pathogenesis of HPNGC.

The strength of this cohort included that large sample size and the long follow-up periods which provided adequate power for us to detect the association between *H*. *pylori* status and prognosis. More importantly, we used three different models to define the *H*. *pylori* status in patients with atrophic gastritis in whom both the serology and histology showed negative for *H*. *pylori*. We demonstrated that negative *H*. *pylori* infection is a worse prognostic factor by using the pepsinogen method. However, there are still some limitations of this study. Firstly, this was a dynamic cohort in which patients with gastric cancer were enrolled during different time periods. The treatments, including the adjuvant chemotherapy regimens and surgical procedures might change over time. However, the major operators of the surgical procedures were the same and total and subtotal gastrectomy were still the standard surgical procedures in our hospital during this time period. Besides, the proportions of HPNGC and HPPGC were not significantly different during the 10 year periods. Secondly, patients taking proton pump inhibitor (PPI), which might affect the sensitivity of histology for detecting *H*. *pylori*, were not excluded. However, serology test for which the sensitivity was less affected was also used in the present study. Thirdly, the correction of multiple comparisons was not done in our subgroup analyses. However, our design was not an exploratory study. Fourthly, decreased antibody levels after H. pylori eradication or the elder age might lead to false negative serology results[44]. We categorized the patients with negative serology test and serologic atrophic gastritis which might be caused by past H. pylori infection into the positive H. pylori status. Also, the serology tests of H. pylori have shown good sensitivity and specificity to detect H. pylori infection[44]. However, it was still our limitation that the H. pylori status should be more accurately assessed with additional methods. Fifthly, we could not collect gastric cancer patients from the Surgery Department and the Oncology Department in National Taiwan University Hospital but only from the Gastroenterology Department. However, we collected our patients completely from the Gastroenterology Department with a very low loss follow up rate (2.4%) and a similar demographic result between participates and non-participates (S1 Table).

In conclusion, we found distinct characteristics of patients with HPNGC compared to HPPGC, including more diffuse type cancer, higher proportion of the proximal tumor location, younger disease onset, and more stage IV disease at the time of diagnosis. The absence of *H*. *pylori i*nfection was an independent poor prognostic factor using different definitions of *H*. *pylori* status, particularly in patients with stage I, II and III disease. These findings collectively lend to support that *H*. *pylori* negative gastric cancer is a distinct disease entity with a more aggressive behavior in the disease progression.

## Supporting Information

S1 Table The characteristics of gastric cancer patients. The demographic result between participates and non-participates was similar.
(PDF)
