# Supplementary material for: Distinct Clinicopathological Features and Prognosis of Helicobacter pylori Negative Gastric Cancer
Source: PLoS One. 2017 Feb 2;12(2):e0170942. doi: 10.1371/journal.pone.0170942 (PMC5289528; doi:10.1371/journal.pone.0170942)
Supplement: S1 Table — The demographic result between participates and non-participates was similar. (PDF) [file pone.0170942.s001.pdf]

Characteristics of gastric cancer patients

	Participate (N=567)	Non participate (N=14)	P value
Age at diagnosis	62.32 +/- 14.25	63.50 +/- 16.15	0.76
Gender			0.45
Men	349(61.5%)	10(71.4%)	
Women	218(38.5%)	4(28.6%)	
H. pylori status			0.35
Negative	75(13.2%)	3(21.2%)	
Positive	492(86.8%)	11(78.8%)	
Tumor location			0.42
Proximal location	79(13.9%)	3(21.4%)	
Distal location	488(86.1%)	11(78.5%)	
Resection modality			0.35
ESD	7 (1.7 %)	0(0%)	
Subtotal gastrectomy	309(77.8%)	9(64.2%)	
Total gastrectomy	81(20.4%)	5(35.8%)	
Histological analysis			
Lauren classification			0.58
Diffuse type	211(37.2%)	5(35.7%)	
Intestinal type	230(40.5%)	7(50.0%)	
Mixed type	126(22.2%)	2 (14.3%)	
Invasive depth			0.41
T1	114(20.9%)	3(21.4%)	
T2	47(8.6)	3(21.4%)	
T3	112(20.6%)	2(14.2%)	
T4	270(49.7)	6(42.8%)	
Nodal metastasis			0.07
N0	180(33.2%)	8(57.1%)	
N1	73(13.4%)	3(21.4%)	
N2	129(23.8%)	3(21.4%)	
N3	160(29.5%)	0(0%)	
Stage (AJCC 7th)			0.12
I	126(23.8%)	3(21.4%)	
II	71(13.4%)	5(35.8%)	

III	173(32.7%)	3(21.4%)
IV	159(30.1%)	3(21.4%)
